# Aging Disrupts the Circadian Patterns of Protein Expression in the Murine Hippocampus

**DOI:** 10.3389/fnagi.2019.00368

**Published:** 2020-01-15

**Authors:** Paula Adler, Cheng-Kang Chiang, Janice Mayne, Zhibin Ning, Xu Zhang, Bo Xu, Hai-Ying Mary Cheng, Daniel Figeys

**Affiliations:** ^1^Shanghai Institute of Materia Medica–University of Ottawa Joint Research Centre on Systems and Personalized Pharmacology, University of Ottawa, Ottawa, ON, Canada; ^2^Ottawa Institute of Systems Biology, University of Ottawa, Ottawa, ON, Canada; ^3^Department of Biochemistry, Microbiology and Immunology, University of Ottawa, Ottawa, ON, Canada; ^4^Department of Biology, University of Toronto Mississauga, Mississauga, ON, Canada; ^5^Department of Cell and Systems Biology, University of Toronto, Toronto, ON, Canada; ^6^Canadian Institute for Advanced Research, Toronto, ON, Canada

**Keywords:** aging, circadian, proteomics, hippocampus, mass spectrometry, neurodegenerative diseases

## Abstract

Aging is associated with cognitive decline and dysregulation of the circadian system, which modulates hippocampal-dependent memory as well as biological processes underlying hippocampal function. While circadian dysfunction and memory impairment are common features of aging and several neurodegenerative brain disorders, how aging impacts the circadian expression patterns of proteins involved in processes that underlie hippocampal-dependent memory is not well understood. In this study, we profiled the hippocampal proteomes of young and middle-aged mice across two circadian cycles using quantitative mass spectrometry in order to explore aging-associated changes in the temporal orchestration of biological pathways. Of the ∼1,420 proteins that were accurately quantified, 15% (214 proteins) displayed circadian rhythms in abundance in the hippocampus of young mice, while only 1.6% (23 proteins) were rhythmic in middle-aged mice. Remarkably, aging disrupted the circadian regulation of proteins involved in cellular functions critical for hippocampal function and memory, including dozens of proteins participating in pathways of energy metabolism, neurotransmission, and synaptic plasticity. These included processes such as glycolysis, the tricarboxylic acid cycle, synaptic vesicle cycling, long-term potentiation, and cytoskeletal organization. Moreover, aging altered the daily expression rhythms of proteins implicated in hallmarks of aging and the pathogenesis of several age-related neurodegenerative brain disorders affecting the hippocampus. Notably, we identified age-related alterations in the rhythmicity of proteins involved in mitochondrial dysfunction and loss of proteostasis, as well as proteins involved in the pathogenesis of disorders such as Alzheimer’s disease and Parkinson’s disease. These insights into aging-induced changes in the hippocampal proteome provide a framework for understanding how the age-dependent circadian decline may contribute to cognitive impairment and the development of neurodegenerative diseases during aging.

## Introduction

Circadian regulation of various physiological and behavioral processes is critical to maintaining homeostasis and organismal health (Bass and Lazar, 2016; Chaix et al., 2016). In mammals, the suprachiasmatic nucleus (SCN) acts as a central pacemaker to entrain an organism’s internal clock using environmental cues such as the daily light/dark cycle, and in turn synchronizes peripheral oscillators located in other brain regions and organs (Liu et al., 2007; Dibner et al., 2010). Circadian disturbances occur during aging and are associated with cognitive decline and several brain disorders, including neurodegenerative diseases (Kondratov, 2007; Kondratova and Kondratov, 2012; Musiek and Holtzman, 2016). Moreover, the circadian clock influences processes involved in hallmarks of aging, underscoring its significance in maintaining physiological integrity (Kondratov, 2007; Lopez-Otin et al., 2013; Fonseca Costa and Ripperger, 2015; Chaix et al., 2016). Disruption of the temporal coordination of clock-controlled processes may therefore contribute to functional decline during aging (Kondratov, 2007; Hood and Amir, 2017). Furthermore, given the interplay between the circadian system and the aging process, and that aging is a major risk factor for several neurodegenerative diseases, the effects of aging on circadian rhythms could have important implications for the pathogenesis of these disorders (Kondratov, 2007; Musiek and Holtzman, 2016). Yet, how aging affects the circadian orchestration of biological processes in the brain remains largely unexplored (Hatanaka et al., 2017).

Several molecular and cellular mechanisms underlying synaptic plasticity and hippocampal function, as well as hippocampal-dependent memory itself, have previously been shown to be clock-regulated and disrupted by clock dysregulation (Smarr et al., 2014; Hannou et al., 2018; Snider et al., 2018). Circadian clocks modulate the expression or activity of diverse proteins involved in processes contributing to hippocampal function, such as neurotransmission, long-term potentiation (LTP), and energy metabolism (Chiang et al., 2017; Hannou et al., 2018; Greco and Sassone-Corsi, 2019). These include proteins such as calcium/calmodulin-dependent protein kinase II (CaMKII), glycogen synthase kinase 3β (GSK3β), the GluA1 AMPA receptor (AMPAR) subunit, synaptic vesicle glycoprotein 2A (SV2A), and isocitrate dehydrogenase (IDH) (Chiang et al., 2014, 2017; Neufeld-Cohen et al., 2016; Hannou et al., 2018; Snider et al., 2018). Moreover, circadian dysfunction and cognitive decline are common features of aging and multiple age-related neurodegenerative disorders, and accumulating evidence suggests that aging- and disease-related circadian disruption contributes to memory impairment (Kondratova and Kondratov, 2012; Musiek and Holtzman, 2016). However, the mechanisms underlying the association between declines in circadian function and memory during aging remain elusive. The identification of age-related changes in the circadian regulation of processes underlying memory function in the hippocampus might therefore open new avenues to correct dysregulated rhythms and thereby prevent or reverse cognitive decline.

Systems biology approaches are uniquely positioned to provide insight into the ubiquitous role of the circadian clock in physiology and to identify links among diverse temporally regulated processes (Hughes et al., 2017; Millius and Ueda, 2017). We have previously shown that the clock regulates key biological processes at the proteomic and phosphoproteomic levels in the SCN and hippocampus of young mice (Chiang et al., 2014, 2017). However, the impact of aging on the hippocampus has not yet been investigated at the global proteomic level from a circadian perspective. In this study, we compared young and middle-aged mice using a quantitative mass spectrometry (MS)-based approach to profile the hippocampal circadian proteome across two consecutive days, in order to dissect changes in the temporal orchestration of biological pathways during aging. We show that aging disrupts the circadian regulation of proteins involved in cellular functions critical for hippocampal function and memory, notably energy metabolism, neurotransmission, and synaptic plasticity. Furthermore, aging altered the daily expression rhythms of proteins implicated in various processes linked to neurodegenerative diseases as well as hallmarks of aging, such as mitochondrial dysfunction and loss of proteostasis. Collectively, our findings provide further evidence supporting the contribution of the age-dependent circadian decline to the development of neurodegenerative diseases and cognitive deterioration over time.

## Materials and Methods

### Animals and Tissue Collection

Male C57BL/6J mice were purchased from the Jackson Laboratory (Bar Harbor, ME, United States; Stock #000664) and aged to 9–10 weeks (young group) or 44–52 weeks (middle-aged group). Mice were group housed in polycarbonate cages with *ad libitum* access to food and water and entrained to a 12-h light:12-h dark (LD) schedule (lights on at 6:00 a.m., lights off at 6:00 p.m.) from 5 weeks of age (young group) or 28 weeks of age (middle-aged group) before being transferred to constant darkness (DD). After 2 days in constant darkness, mice were sacrificed at 4-h intervals over 2 days starting at circadian time (CT) 2 on the third day of DD, where CT was defined by the zeitgeber time (ZT) of the previous LD schedule (Chiang et al., 2014, 2017). Sample sizes were as follows: three mice per age group were sacrificed at each CT, except for CT18 and 22 (four mice per age group at each CT), CT42 (four young mice and two middle-aged mice), and CT46 (four young mice). Mice were sacrificed by cervical dislocation under dim red light and the hippocampi were quickly excised. Tissues were immediately flash frozen in liquid nitrogen and stored at −80°C until further processing. All animal experiments were conducted at the Ottawa Hospital Research Institute and approved by the University of Ottawa Animal Care Committee in compliance with institutional and Canadian Council on Animal Care guidelines.

### Proteomic Analysis of Hippocampal Tissues

Protein extracts from hippocampal tissues of individual mice were obtained by homogenization in lysis buffer using a pellet pestle and sonication (three 10 s pulses with 30 s on ice between each pulse). The lysis buffer contained 4% (w/v) sodium dodecyl sulfate (SDS) in 50 mM ammonium bicarbonate (ABC; pH 8.2) supplemented with complete protease and phosphatase inhibitor cocktails (Roche; Mississauga, ON, Canada). Protein concentrations were determined using the DC Protein Assay (Bio-Rad; Mississauga, ON, Canada), and hippocampal lysates were loaded onto 30-kDa molecular weight cutoff Microcon filters (MilliporeSigma; Oakville, ON, Canada). Proteins were reduced by incubating samples with 20 mM dithiothreitol (DTT; MilliporeSigma; Oakville, ON, Canada) for 30 min at 37°C with agitation (245 rpm) and subsequently alkylated with 20 mM 2-iodoacetamide (IAA; MilliporeSigma; Oakville, ON, Canada) for 30 min in darkness at room temperature. Protein digestion was performed by incubation with 40:1 (w/w, protein:enzyme) trypsin (Worthington Biochemical Corporation; Lakewood, NJ, United States) overnight at 37°C with agitation (245 rpm). Prior to strong cation exchange (SCX) fractionation of hippocampal samples, peptides were diluted with 0.1% (v/v) formic acid (FA) and the pH adjusted with trifluoroacetic acid (TFA) to 3.0. Step elution of peptides was performed using in-house-made SCX columns (10-μm SCX beads, Polymer Laboratories) and subsequent addition of buffers (20 mM boric acid, 20 mM phosphoric acid, and 20 mM acetic acid) at pH 5, 6, 8, 10, and 12. Samples were desalted using in-house-made C18 desalting cartridges (C18 beads: ReproSil-Pur C18-AQ, 10 μm; Dr. Maisch GmbH, Germany) and dessicated using a SpeedVac prior to being resuspended in 0.1% (v/v) FA for liquid chromatography tandem MS (LC–MS/MS) analysis.

### LC–MS/MS Analysis

Four microliters of resuspended peptides (equivalent to 2 μg of proteins) from each sample were analyzed by an online reverse-phase LC–MS/MS platform consisting of an Eksigent NanoLC 425 System (AB SCIEX) coupled with an Orbitrap Elite mass spectrometer (Thermo Fisher Scientific, San Jose, CA, United States) via a nano-electrospray source. Prior to MS analysis, peptide mixtures were separated by reverse-phase chromatography using an in-house packed ReproSil-Pur C18-AQ column (75 μm internal diameter × 15 cm, 1.9 μm, 200 Å pore size; Dr. Maisch GmbH, Germany) over a 120-min gradient of 5–30% buffer B [acetonitrile (ACN) with 0.1% (v/v) FA] at a flow rate of 300 nl/min. The Orbitrap Elite instrument was operated in the data-dependent mode to simultaneously measure survey scan MS spectra (350–1,800 *m*/*z*, *R* = 60,000 defined at *m*/*z* 400). Up to the 20 most intense peaks were isolated and fragmented with collision-induced dissociation (CID). System controlling and data collection were carried out using Xcalibur software version 2.2 (Thermo Scientific).

### Mass Spectrometry Data Processing

Mass spectrometry raw files were processed with MaxQuant (version 1.5.2.8) using the integrated Andromeda search engine and UniProt FASTA database from mouse (*Mus musculus*; 2013_05). The search included variable modifications for methionine oxidation (M) and acetylation (protein N-term) as well as fixed modification for carbamidomethylation (C). Trypsin/P was set as the cleavage specificity with up to two missed cleavages allowed. The false discovery rate (FDR) cutoffs were set at 0.01 at the peptide and protein levels and the minimum peptide length was set at 7. Identification across different replicates and adjacent fractions was achieved by enabling the “match between runs” option with a matching time window of 5 min.

### Bioinformatic and Statistical Analyses

Initial bioinformatic analysis was performed with Perseus (version 1.5.5.3). Following logarithmic (log_10_) transformation of label-free quantification (LFQ) intensities, the raw proteomic dataset was filtered to include only proteins quantified in a minimum of two biological replicates per time point in either young or middle-aged mice. Using these filtered datasets, circadian rhythmicity in protein abundance over the 12 CTs was determined using the Perseus periodicity algorithm (period = 23.6 h) (Robles et al., 2014) with *q*-value < 0.25 (Mauvoisin et al., 2014). Heatmaps displaying temporal expression profiles of circadian proteins ordered by phase were generated using the logarithmized LFQ intensities after *z*-score normalization. Gene Ontology (GO) and Kyoto Encyclopedia of Genes and Genomes (KEGG) pathway functional annotations and enrichment analyses were implemented using DAVID (version 6.8; one-sided Fisher’s exact test *p* ≤ 0.05 relative to the backgrounds of accurately quantified proteins in our datasets was considered significant) in order to assess changes in GO biological processes, GO cellular components, and KEGG pathways. Protein–protein interaction networks were created using the STRING database (Szklarczyk et al., 2015) (confidence score cutoff = 70%) and visualized with Cytoscape (version 3.4.0) to include the phases and *q*-values of rhythmic proteins.

### Data Availability Statement

The mass spectrometry proteomics data have been deposited to the ProteomeXchange Consortium via the PRIDE partner repository with the dataset identifier PXD013364. Note that a stable isotope labeling by amino acids in cell culture (SILAC) spike-in was introduced during sample preparation but not used for quantification.

## Results

### Aging Disrupts the Hippocampal Circadian Proteome

**FIGURE 1:**
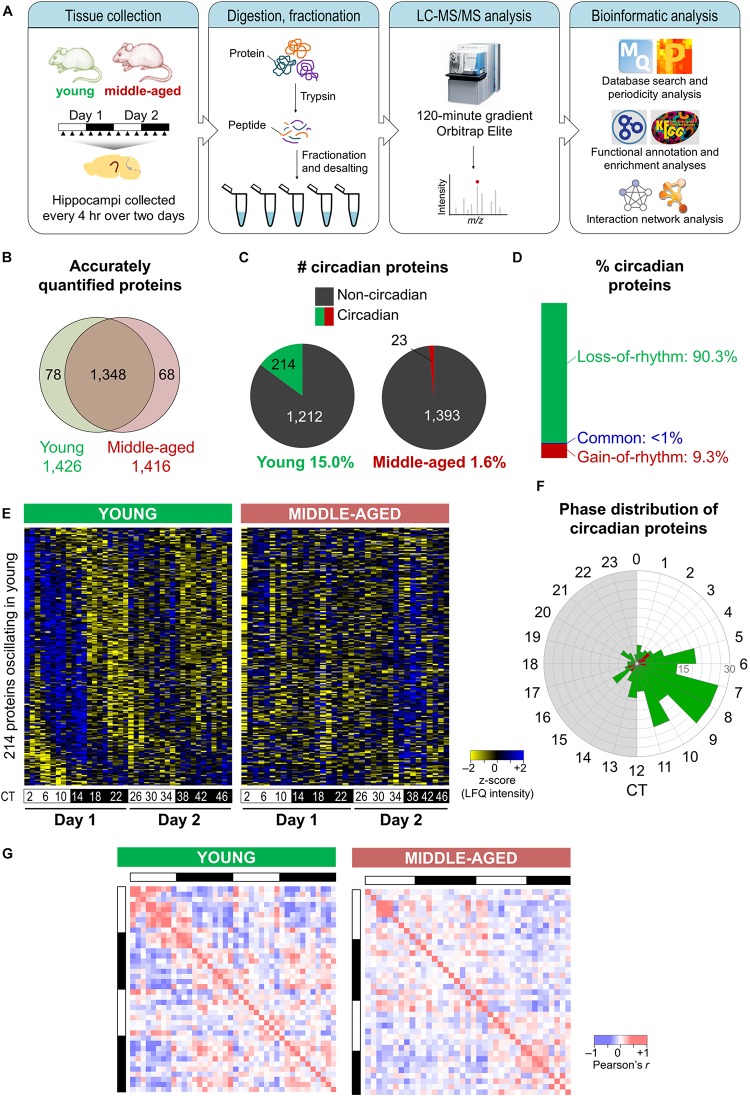
Aging disrupts the hippocampal circadian proteome. **(A)** Experimental design and workflow of the MS-based analysis of proteins extracted from hippocampal tissues of young (9–10 weeks old) and middle-aged (44–52 weeks old) C57BL/6J mice. Samples were collected every 4 h over 2 days, and proteins extracted from tissues of individual mice were digested with trypsin, fractionated, and analyzed by an Orbitrap Elite mass spectrometer. **(B)** Proteome coverage: Venn diagram displaying the number of proteins quantified in at least two biological replicates per time point in young or middle-aged mice and overlap between ages. **(C)** Circadian proteins detected in young or middle-aged mice using the Perseus periodicity algorithm (period = 23.6 h; *q*-value < 0.25). **(D)** Percent distribution of circadian proteins based on changes in rhythmicity during aging. **(E)** Heatmaps displaying *z*-score normalized abundances (log_10_ LFQ intensities) of circadian proteins detected in young mice and their temporal expression profiles in young mice (left) and middle-aged mice (right). **(F)** Phase distribution of circadian proteins detected in young mice (green) or middle-aged mice (red). **(G)** Correlation heatmaps across 48 h in young mice (left) and middle-aged mice (right) for circadian proteins detected in young mice. Pearson correlation coefficients are shown as red (positive) or blue (negative).

To examine how aging alters the regulation of rhythmic processes in the hippocampus, we used a quantitative MS-based approach to analyze total protein extracts from hippocampal tissues harvested from young and middle-aged mice over two consecutive circadian cycles (see the section “Materials and Methods”; Figure 1A). Samples were processed individually to yield three to four biological replicates at each of the 12 time points for each age group, and relative protein abundances were determined using LFQ. This MS-based analysis identified a total of 4,433 proteins, of which 1,426 and 1,416 were quantified in a minimum of two biological replicates at each time point in the hippocampus of young mice and middle-aged mice, respectively (Figure 1B). We used these stringently filtered datasets of reliably quantified proteins for downstream analysis (Supplementary Data 1, 2).

Proteins displaying circadian oscillations in their abundances were identified using the Perseus periodicity analysis algorithm (period = 23.6 h) (Robles et al., 2014) and a FDR value cutoff of 0.25 (Mauvoisin et al., 2014) in young and middle-aged mice. Abundance for 236 proteins changed as a function of circadian timing at either age (Supplementary Data 3, 4). As expected, the core circadian clock proteins were not accurately quantified in our datasets due to their low abundance in total protein extracts (Millius and Ueda, 2017). A total of 214 proteins displayed circadian rhythmicity in the hippocampus of young mice (15.0% of reliably quantified proteins), while in middle-aged mice a total of 23 rhythmic proteins were identified (1.6% of reliably quantified proteins) (Figure 1C). Overall, there were fewer rhythmic proteins identified in the middle-aged group, reflective of the age-related decline in the circadian system. Strikingly, ∼90% of circadian proteins detected at either age displayed a loss of rhythmicity during aging (i.e., oscillated exclusively in young mice) (Figure 1D), suggesting that aging is associated with widespread disruption in the temporal regulation of cellular functions in the hippocampus.

Although most age-related changes involved a loss of rhythmicity in middle-aged mice, ∼9% of all circadian proteins detected did not oscillate in young mice but gained rhythmicity during aging (Figure 1D). These included proteins involved in specific processes known to play roles in aging, such as apoptosis and the cellular stress response (Supplementary Figure 1). Consistent with our results, two previous studies have identified sets of transcripts that gained rhythmicity during aging in the human prefrontal cortex and in heads of *Drosophila melanogaster*, notably genes involved in stress response functions (Chen et al., 2016; Kuintzle et al., 2017). Moreover, proteins that have been linked to neurodegenerative diseases were among those displaying rhythmic abundances in the hippocampus of middle-aged mice (Supplementary Table 1). For instance, histone deacetylase 1 (HDAC1) as well as one of its target proteins, histone H3, gained rhythmicity during aging. Interestingly, histone acetylation regulates memory function as well as transcription of clock genes, and age-related changes in hippocampal-dependent memory have previously been linked to epigenetic modulation of the clock gene *Per1* through HDAC3 (Kwapis et al., 2018). Thus, aging might result in altered circadian epigenetic regulation of clock genes and other genes that affect hippocampal function.

Rhythmic proteins displayed a variety of temporal abundance profiles in the hippocampus of young mice (left panel in Figure 1E), with the phases of peak expression clustering in the afternoon (Figure 1F), while in middle-aged mice all but one of these proteins were no longer detected as oscillating (right panel in Figure 1E). Furthermore, aging resulted in loss of positive and negative correlations among rhythmic proteins detected in young mice across the day/night cycle (Figure 1G). Together, our findings indicate that there are widespread aging-induced changes in the daily patterns of protein expression in the hippocampus.

### Age-Related Changes in the Circadian Regulation of Biological Functions

**FIGURE 2:**
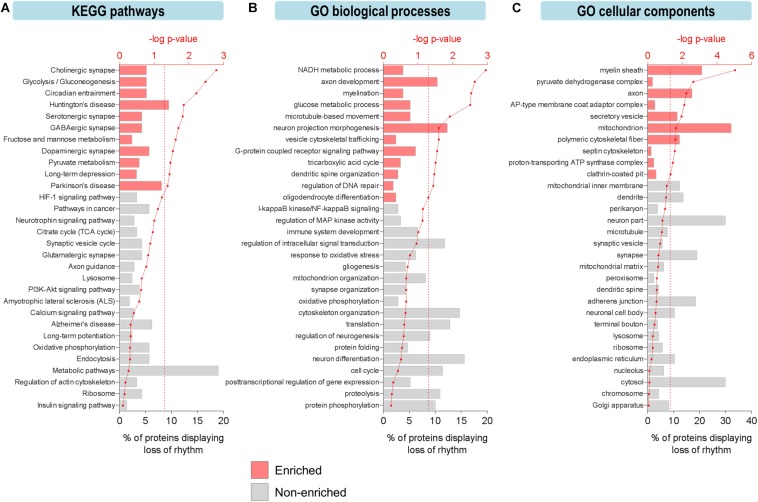
Aging leads to loss of circadian oscillations in specific biological functions in the hippocampus. **(A–C)** Functional analysis of proteins displaying loss of circadian rhythmicity with aging. Enriched ontology terms corresponding to **(A)** KEGG pathways, **(B)** GO biological processes, and **(C)** GO cellular components are shown with colored bars (*p* ≤ 0.05, Fisher’s exact test relative to background of accurately quantified proteins in our dataset).

To explore the functional relevance of these age-related changes, we examined biological pathways and processes using KEGG and GO analyses to identify functional terms over-represented among proteins displaying loss of rhythmicity in abundance during aging (Figure 2). Overall, pathways involved in basic cellular metabolism, circadian entrainment, synaptic function, and neurodegenerative diseases were among those enriched (Figure 2A). Several enriched processes have previously been characterized as circadian in young mammalian models, including the tricarboxylic acid (TCA) cycle, dendritic spine organization, and regulation of DNA repair (Smarr et al., 2014; Chaix et al., 2016; Neufeld-Cohen et al., 2016). Biological processes involved in neurotransmission, synaptic plasticity, mitochondrial function, redox homeostasis, and proteostasis were also highly represented among proteins displaying age-related loss of circadian rhythmicity (Figure 2B).

Furthermore, protein–protein interaction network analysis of proteins displaying loss of rhythmicity in the hippocampus during aging revealed interactions among proteins involved in energy metabolism, mitochondrial function, cytoskeletal organization, translation, and G-protein signaling (Supplementary Figure 2). Given that many of these affected processes are critical for normal hippocampal function and have been implicated in neurodegenerative brain disorders, their altered circadian regulation might contribute to aging- and disease-associated decline in hippocampal function and memory. For instance, dysfunction of G-protein-coupled neurotransmission occurs in the brain during aging and may predispose older individuals to developing age-related neurodegenerative disorders (Mattson and Arumugam, 2018).

The effects of aging on rhythmic biological functions were complemented by results from a GO analysis of cellular components. In young mice, rhythmic proteins participated in diverse processes involved in the structure and function of various intracellular organelles, with the mitochondrion and cytoskeleton being two major sites of circadian regulation (Figure 2C). Proteins localized to the mitochondrion (including the mitochondrial inner membrane, ATP synthase complex, and pyruvate dehydrogenase complex) and synapse (including synaptic vesicles, terminal boutons, and septin cytoskeleton) displayed loss of circadian oscillations in abundance, reflective of the pathways related to mitochondrial energy metabolism, neurotransmission, and synaptic plasticity (Figures 2A,B).

Interestingly, some of the most highly enriched GO functional terms included myelination, myelin sheath, and oligodendrocyte differentiation (Figures 2B,C), which might suggest another mechanism through which aging disrupts cognitive function. Consistent with this hypothesis and our results showing that proteins involved in these functions peak during the daytime in young mice, it has previously been reported that genes involved in myelination and promoting proliferation of oligodendrocyte precursor cells (OPCs) are transcribed preferentially during sleep in mice (Bellesi et al., 2013). Furthermore, OPCs demonstrate daily rhythms in proliferation in the hippocampus of young mice (Matsumoto et al., 2011) and sleep loss has been shown to disrupt myelin formation (Bellesi et al., 2018). While previous work has shown that myelination is compromised in the aged human brain and especially in people with cognitive deficits (Mattson and Arumugam, 2018), our results suggest that age-related changes in the circadian regulation of myelination may also contribute to cognitive decline during aging via neural circuit dysfunction and impaired communication between different brain regions.

We also noted age-related changes in rhythmic abundances of multiple proteins involved in regulating protein phosphorylation (Figure 2B), indicating that temporal control of post-translational modifications such as phosphorylation may be altered and associated with cognitive decline during aging. Interestingly, we found that the phases of peak expression of circadian proteins clustered in the afternoon, at the same time as the peak in rhythmic phosphoproteins we have previously reported (Chiang et al., 2017). Moreover, aging-associated changes in the daily expression profiles of kinases such as CaMKII and GSK3β (Supplementary Figure 3) might contribute to the age-related declines in hippocampal-dependent memory and local clock function (Kon et al., 2014; Besing et al., 2017). GSK3β, which phosphorylates and regulates BMAL1 and REV-ERBα (Reischl and Kramer, 2011), has previously been shown to play important roles in modulating synaptic plasticity as well as the molecular clock in the hippocampus of young mice (Besing et al., 2017). Wang et al. (2017) previously reported that GSK3β peaks in nuclear activity/abundance in the livers of young mice during the day, consistent with our hippocampal study. In addition, other proteins involved in clock function displayed a loss of rhythmicity in the hippocampus of middle-aged mice (Supplementary Figure 3), highlighting the age-dependent decline in local clock function at the molecular level. Taken together, our findings indicate that there is widespread disruption in the circadian regulation of biological processes and pathways critical for normal hippocampal function during aging.

### Aging Disrupts the Circadian Regulation of Energy Metabolism

Dysregulated energy metabolism is a hallmark of brain aging and its exacerbation underlies the molecular pathogenesis of several age-related neurodegenerative disorders, including Alzheimer’s disease (AD), amyotrophic lateral sclerosis (ALS), Parkinson’s disease (PD), and Huntington’s disease (HD) (Camandola and Mattson, 2017; Mattson and Arumugam, 2018). We therefore further examined the effects of aging on the circadian regulation of energy metabolism in the hippocampus, focusing on proteins involved in glucose metabolism as well as the downstream pyruvate metabolism, TCA cycle, and oxidative phosphorylation pathways.

**FIGURE 3:**
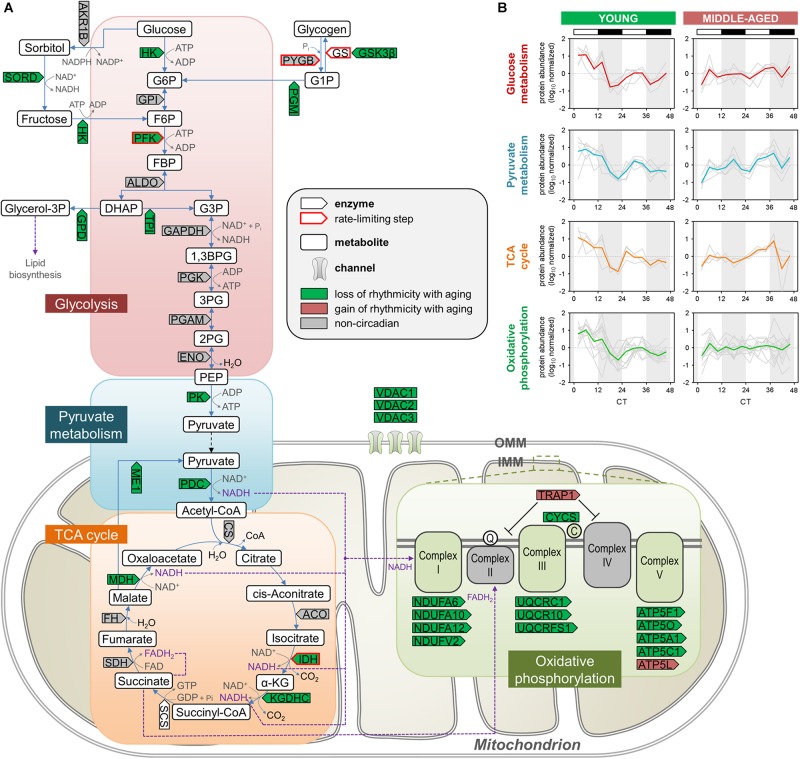
Aging disrupts the circadian regulation of proteins involved in energy metabolism in the hippocampus. **(A)** Schematic depiction of proteins involved in energy metabolism, including glycolysis, pyruvate metabolism, the TCA cycle, and oxidative phosphorylation. Proteins that displayed loss or gain of circadian rhythmicity in abundance during aging are highlighted in color. Proteins that were reliably quantified, but not detected as circadian at either age, are shown in gray. **(B)** Temporal abundance profiles of rhythmic proteins identified in young mice involved in energy metabolism.

The relative enrichment among proteins displaying loss of rhythmicity during aging of pathways such as glycolysis, pyruvate metabolism, and the TCA cycle (Figures 2A,B) is consistent with previous studies from our lab and others demonstrating that several components of these pathways exhibit diurnal rhythms in protein abundance in the brain as well as peripheral organs of young mice (Chiang et al., 2014, 2017; Neufeld-Cohen et al., 2016). We found over 20 proteins participating in critical energy metabolism pathways that displayed loss of rhythmicity in the hippocampus during aging (Figure 3A). In young mice, these rhythmic proteins peaked in a coordinated manner during the day (Figure 3B), and at the same time that circadian proteins involved in oxidative phosphorylation peak in the SCN of young mice (Chiang et al., 2014). Interestingly, many of the rhythmic proteins catalyzing steps in glycolysis and the TCA cycle in young mice are NAD^+^-dependent enzymes, suggesting a potential mechanism by which circadian oscillations in NAD^+^ could couple energy production in the brain to the daily light/dark cycle (Peek et al., 2013). Importantly, phosphofructokinase (PFK) and the regulatory subunit of isocitrate dehydrogenase 3 (IDH3) were rhythmic in young mice but not middle-aged mice. Given that these enzymes catalyze the rate-limiting steps of glycolysis and the TCA cycle, respectively, aging may have a significant impact on the dynamics of these pathways and therefore energy production in the hippocampus. These results suggest that the dysregulation of energy metabolism that occurs in the brain during aging extends to its temporal regulation, and disrupted circadian rhythms in energy metabolism pathways might contribute to age-related impairments in hippocampal function and memory.

### Aging Disrupts the Circadian Regulation of Synaptic Structure and Function

Circadian modulation of hippocampal-dependent memory at the molecular level can occur through clock regulation of multiple neuronal and synaptic components, including synaptic vesicle proteins, receptors, transporters, and intracellular signaling cascades (Hannou et al., 2018; Rawashdeh et al., 2018; Snider et al., 2018). Thus, we were interested in examining the impact of aging on the circadian rhythmicity of proteins involved in synaptic plasticity and function in the hippocampus, given that changes in the regulation of these processes may contribute to age-related memory impairment.

**FIGURE 4:**
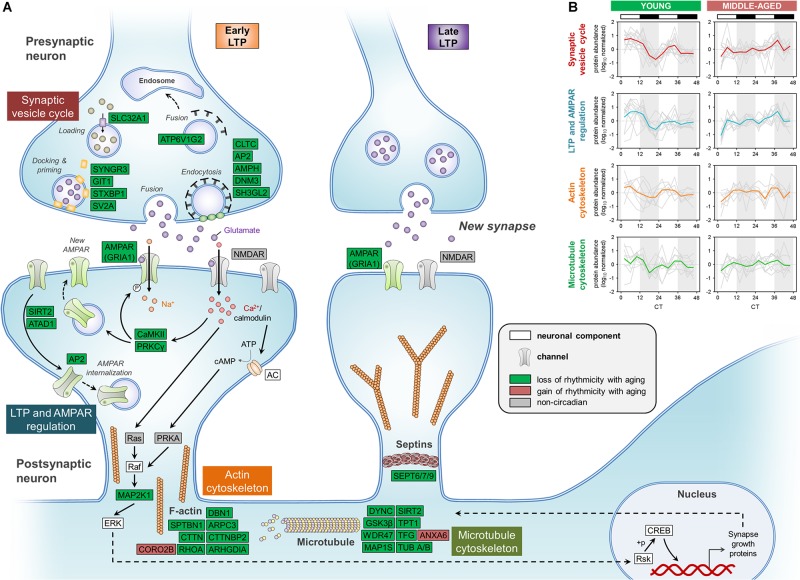
Aging disrupts the circadian regulation of proteins involved in synaptic function in the hippocampus. **(A)** Schematic depiction of proteins involved in synaptic structure and function, including the synaptic vesicle cycle, LTP and AMPAR regulation, and the cytoskeleton. Proteins that displayed loss or gain of circadian rhythmicity in abundance during aging are highlighted in color. Proteins that were reliably quantified, but not detected as circadian at either age, are shown in gray. **(B)** Temporal abundance profiles of rhythmic proteins identified in young mice involved in synaptic structure and function.

We first examined the effects of aging on synaptic vesicle cycling, which has previously been shown to be rhythmic and important for circadian gene expression in the SCN (Deery et al., 2009). Strikingly, middle-aged mice displayed a loss of circadian oscillations in the abundances of over 10 proteins involved in this pathway, which were rhythmic in the hippocampus of young mice (Figure 4A). These included syntaxin binding protein 1 (also known as Munc18-1), SV2A, and endophilin A1, which we and others have previously shown to be clock-regulated at the protein or mRNA level in the brains of young mice (Chiang et al., 2014; Hannou et al., 2018). Interestingly, we also found that the GluA1 AMPAR subunit, along with other proteins regulating AMPAR phosphorylation and trafficking, such as CaMKII and protein kinase C γ, displayed loss of rhythmicity in the hippocampus during aging. Previous work has demonstrated that glutamate receptor trafficking is regulated by clock-gated signaling pathways, in line with our results (Snider et al., 2018). In addition, multiple cytoskeletal components and regulators lost rhythmicity in middle-aged mice, including actin regulators such as RHOA and Rho GDP-dissociation inhibitor (RhoGDI), whose interaction is known to be clock-regulated (Ma et al., 2018). Several proteins involved in the organization of microtubules and the septin cytoskeleton were also found to display loss of circadian oscillations in the hippocampus of middle-aged mice, suggesting that aging could lead to disruption of synaptic plasticity through multiple neuronal cytoskeleton components given that synaptic plasticity is affected by cytoskeletal remodeling (Gordon-Weeks and Fournier, 2014).

While circadian rhythmicity of proteins involved in synaptic structure and function was largely lost in middle-aged mice, these proteins peaked in a coordinated manner during the day in the hippocampus of young mice (Figure 4B), consistent with previous work demonstrating that processes supporting synaptic plasticity peak during sleep in mice (Eckel-Mahan et al., 2008). Thus, the age-related dampening in rhythmicity of proteins involved in the synaptic vesicle cycle, LTP and AMPAR regulation, and cytoskeleton organization might lead to compromised synaptic structure and function in the hippocampus of middle-aged mice, which could in turn result in impaired hippocampal-dependent memory during aging.

### Disruption of Rhythmic Proteins Involved in Hallmarks of Aging and Neurodegenerative Diseases

**FIGURE 5:**
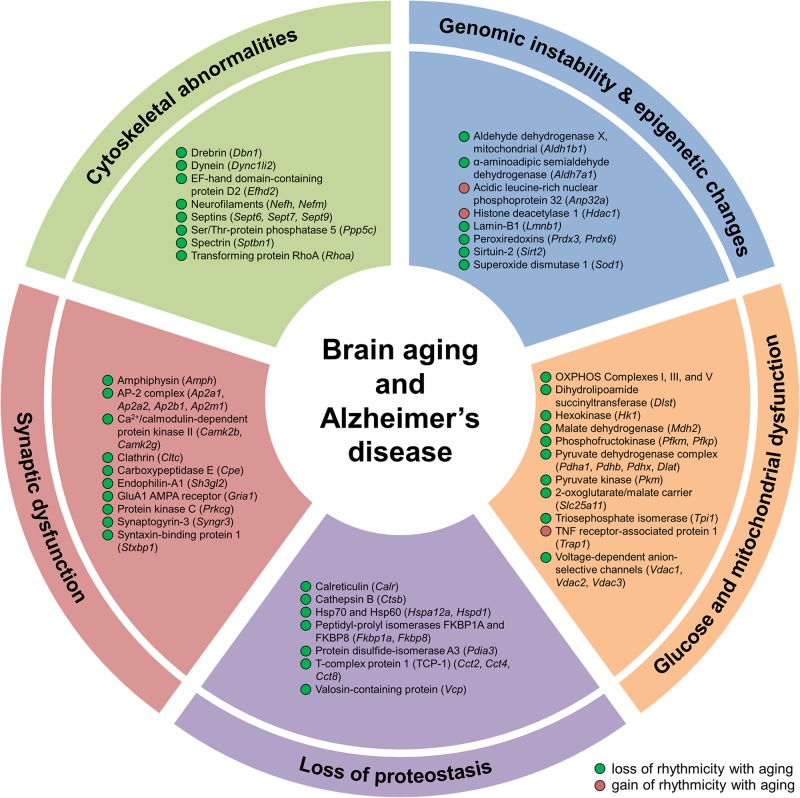
Aging alters hippocampal circadian oscillations of proteins implicated in Alzheimer’s disease and hallmarks of aging. Proteins displaying changes in rhythmicity during aging and involved in hallmarks of aging and Alzheimer’s disease pathogenesis: genomic instability, epigenetic alterations, loss of proteostasis, glucose and mitochondrial dysfunction, synaptic dysfunction, and cytoskeletal abnormalities.

Mitochondrial dysfunction, a hallmark of aging, may contribute to age-associated damage and has been implicated in cognitive impairment as well as the pathogenesis of several neurodegenerative diseases (Lopez-Otin et al., 2013; Mattson and Arumugam, 2018). We were therefore interested in examining whether the age-related decline in efficiency of mitochondrial energy metabolism and antioxidant defense mechanisms extends to their circadian regulation in the hippocampus (Green et al., 2011). As discussed above, circadian rhythms of mitochondrial proteins involved in energy metabolism were particularly affected during aging, with over 20 proteins displaying age-related loss of rhythmicity (Figure 3). These proteins included IDH3, which catalyzes the rate-limiting step of the TCA cycle, as well as several subunits belonging to electron transport chain complexes (Figures 3, 5). In young mice, proteins involved in mitochondrial respiration peaked during the day along with many antioxidant enzymes, notably superoxide dismutase 1 (SOD1) and peroxiredoxins (PRDX3 and PRDX6). Interestingly, we found that these and other antioxidant enzymes displayed loss of rhythmic abundances during aging (Figure 5). We also found that all three voltage-dependent anion-selective channels (VDACs) lost their rhythmic expression in middle-aged mice, which could impact both mitochondrial function and synaptic plasticity in the hippocampus (Levy et al., 2003; Figure 5). Thus, aging-induced changes in the circadian rhythms of proteins involved in mitochondrial function and reactive oxygen species (ROS) homeostasis provide additional potential mechanisms through which aging may lead to an increased susceptibility to brain disorders characterized by mitochondrial dysfunction, including neurodegenerative diseases such as AD and PD (Mattson and Arumugam, 2018).

Genomic instability and epigenetic alterations, two primary hallmarks of aging, are linked to clock function through circadian regulation of proteins involved in ROS homeostasis and epigenetic modification. Given that mitochondrial respiration is a major intracellular source of ROS (Mattson and Arumugam, 2018), and that both oxidative phosphorylation and the cellular response to oxidative stress display circadian rhythmicity (Chaix et al., 2016), age-related changes in the temporal control of proteins participating in these two processes (as discussed above) may lead to alterations in the accumulation of genetic damage. Genomic instability can also result from disruption of nuclear lamins, such as lamin B1 (Lopez-Otin et al., 2013). Levels of lamin B1 decline during aging (Lopez-Otin et al., 2013) and exhibited a loss of rhythmicity in the hippocampus of middle-aged mice (Figure 5), suggesting that this disruption could lead to aberrations in the nuclear lamina and contribute to genome instability during aging. We also found age-dependent changes in the rhythmicity of three proteins involved in epigenetic modification, specifically post-translational modification of histones. These include HDAC1, sirtuin 2 (SIRT2), and acidic leucine-rich nuclear phosphoprotein 32 (ANP32A), which are all involved in regulating histone acetylation levels (Figure 5). Age-related changes in the circadian regulation of proteins involved in maintaining genomic and epigenetic stability might therefore result in transcriptional alterations and increased DNA damage, which could in turn stimulate accelerated aging and lead to increased disease risk.

Loss of protein homeostasis, another primary hallmark of aging, is linked to various age-related diseases (Lopez-Otin et al., 2013). While diurnal rhythms in protein synthesis, processing, and degradation have previously been described in several organs in young mammals (Panda et al., 2002; Robles et al., 2014), the impact of aging on the rhythmicity of these processes is less well characterized. We found that the daily abundance profiles of rhythmic proteins participating in protein synthesis, folding, and degradation were altered during aging (Figure 5 and Supplementary Figure 2). These proteins included the molecular chaperones calreticulin, heat shock proteins 60 and 70 (HSP60 and HSP70), and T-complex protein 1 (TCP-1) subunits, as well as valosin-containing protein (VCP), which also regulates ubiquitin-dependent protein degradation (Dai and Li, 2001). Our results suggest that the temporal control of protein homeostasis in the hippocampus is altered during aging, which may in turn impact the core circadian clock transcriptional–translational feedback loop and thus affect oscillations in other local clock-controlled genes (Takahashi, 2017).

To further explore links between age-related disorders and changes in circadian rhythmicity of biological functions during aging, we examined disease-associated pathways containing proteins whose abundance was under circadian regulation in the hippocampus. We found rhythmic proteins associated with the pathogenesis of several age-related neurodegenerative diseases affecting the brain (Supplementary Table 1). In addition to proteins involved in the hallmarks of aging discussed above that are known to play roles in the pathogenesis of AD, such as mitochondrial dysfunction and loss of proteostasis, several other proteins displaying age-related changes in rhythmicity in the hippocampus were also associated with AD. These included proteins involved in synaptic dysfunction and cytoskeletal abnormalities, such as synaptic vesicle proteins, cytoskeleton components and regulators, and enzymes that regulate protein phosphorylation (Figure 5 and Supplementary Table 1). Several proteins linked to AD and involved in the synaptic vesicle cycle were found to display loss of rhythmicity in the hippocampus during aging, including amphiphysin, AP-2 complex subunits, clathrin heavy chain, endophilin-A1, synaptogyrin-3, and syntaxin-binding protein 1 (Figures 4, 5 and Supplementary Table 1). Additional proteins involved in synaptic function and dysregulated in AD included the GluA1 AMPAR subunit, CaMKII, and protein kinase C γ, which all displayed loss of rhythmicity during aging (Figure 4 and Supplementary Table 1). Moreover, several cytoskeletal proteins and regulators known to be implicated in AD pathogenesis were found to lose rhythmicity during aging, including neurofilaments, septins, and actin regulators such as drebrin and RHOA (Supplementary Table 1 and Figures 4, 5). Our data indicate that age-dependent changes in circadian regulation of various processes are associated with and might contribute to AD pathogenesis.

While decline in hippocampal function is a prominent feature of AD, it also occurs in several other neurodegenerative disorders, namely PD (Camicioli et al., 2003), HD (Spargo et al., 1993), ALS (Takeda et al., 2009), and frontotemporal dementia (FTD) (Laakso et al., 2000). We were therefore also interested in identifying age-related hippocampal alterations in the rhythmicity of proteins involved in these diseases. We found that several proteins believed to play major roles in the pathogenesis of these diseases displayed changes in circadian regulation in the hippocampus during aging, notably TDP-43, SOD1, and TNF receptor-associated protein 1 (TRAP1) (Supplementary Table 1). TDP-43, which gained rhythmicity during aging, has been identified as the major pathological protein in both ALS and FTD (Mackenzie and Rademakers, 2008). Moreover, SOD1 displayed a loss of rhythmicity during aging, and mutations in *SOD1* and the TDP-43 gene *TARDBP* are known to occur in familial ALS (Millecamps et al., 2010). The mitochondrial chaperone TRAP1, also known as HSP75, gained rhythmicity in the hippocampus of middle-aged mice and has been linked to familial PD arising due to mutations in *PTEN induced putative kinase 1* (*PINK1*) (Pridgeon et al., 2007). Interestingly, the protective role of PINK1 against oxidative stress is mediated through phosphorylation of TRAP1 and inhibition of the mitochondrial release of cytochrome c (Pridgeon et al., 2007), another protein that displayed loss of rhythmicity in the hippocampus during aging (Supplementary Table 1). Taken together, these results provide further evidence for the association of age-related circadian disruption in the brain with the pathogenesis of neurodegenerative diseases. Furthermore, our findings highlight the widespread aging-associated alterations in temporal regulation of biological processes implicated in aging and age-related brain disorders.

## Discussion

Given the regulation of hippocampal physiology and function by the circadian clock, the age-dependent decline of the circadian system may contribute to cognitive decline over time and development of aging-associated neurodegenerative disorders. Previous large-scale studies of circadian rhythms, while mainly restricted to examining rhythmic processes in young model organisms at the transcriptomic level, have greatly contributed to our understanding of the circadian system’s role in modulating various processes in a tissue-specific manner (Zhang et al., 2014; Millius and Ueda, 2017). Proteomic analyses, which incorporate additional mechanisms of circadian regulation at the post-transcriptional level, have been performed on the brain and peripheral organs of young mice to reveal large-scale coordination of biological processes by the clock (Millius and Ueda, 2017). However, how aging modifies clock-controlled processes has only recently begun to be explored at the transcriptomic level in the liver and stem cells of mice (Sato et al., 2017; Solanas et al., 2017), while the effects in the brain are largely unknown. A large-scale characterization of hippocampal circadian rhythms in protein abundances at different ages would therefore provide insight into age-related perturbations in the timing of cellular functions and could facilitate future studies on the molecular mechanisms of aging- and disease-associated cognitive impairment. Further studies are needed to identify age-related alterations in circadian rhythms of protein abundance in peripheral organs as well as other brain regions, particularly the central pacemaker in the SCN.

In this study, we used a quantitative MS-based approach to analyze hippocampal tissues from young and middle-aged mice and to explore the effects of aging on circadian regulation at the proteomic level. Middle-aged mice demonstrate impaired learning and memory (Shoji et al., 2016), indicating that age-dependent hippocampal proteomic alterations might contribute to cognitive decline at this age. We found that there is widespread disruption of the circadian orchestration of protein expression rhythms in the hippocampus during aging, reflective of the age-related decline in the circadian system. Notably, we have shown that aging leads to a loss of temporal coordination of pathways critical for normal hippocampal function, including energy metabolism, neurotransmission, and synaptic plasticity.

Rhythmic proteins involved in energy metabolism and synaptic vesicle cycling peaked in a coordinated manner during the day in young mice, complementing previous work showing that the synaptic vesicle cycle is the main source of activity-driven metabolic demand at synapses (Rangaraju et al., 2014). Importantly, these pathways lost rhythmicity in the hippocampus of middle-aged mice, suggesting that aging is associated with a disruption in the temporal coupling between energy demand and production, which could lead to impaired synaptic function. High rates of energy production in the brain are required to support neuronal and glial activities, with neurons relying on glucose as their main energy source (Camandola and Mattson, 2017). Moreover, reduced hippocampal energy metabolism (particularly glucose metabolism) has been associated with cognitive impairment and AD (Mattson and Arumugam, 2018). Circadian regulation of energy metabolism and synchronization among local brain clocks might contribute to sustaining daily cycles in brain function, such as increases in memory consolidation and synaptic rewiring during sleep (Kyriacou and Hastings, 2010). Synaptic plasticity must be supported during sleep, when consolidation of memories is hypothesized to preferentially occur (Kondratova and Kondratov, 2012). In line with this hypothesis, circadian oscillations in signal transduction events underlying LTP in the hippocampus have previously been shown to peak during the day in young mice, and disruption of these rhythms results in impaired hippocampal-dependent memory (Eckel-Mahan et al., 2008). Thus, disruption of the temporal coordination of energy production, which supports these functions in the hippocampus, might contribute to age-related cognitive impairment.

The progressive functional deterioration that characterizes aging is a primary risk factor for various age-related disorders, including neurodegenerative diseases (Kondratova and Kondratov, 2012). Interestingly, sleep and circadian disturbances are shared clinical features of several neurodegenerative diseases, and a growing body of evidence indicates that disruption of circadian rhythms contributes directly to their pathogenesis (Kondratova and Kondratov, 2012; Musiek and Holtzman, 2016). Given that these disorders involve an exacerbation of aging-associated circadian dysfunction and hallmarks of aging (Mattson and Arumugam, 2018; Musiek and Holtzman, 2016), identifying changes in the daily cycles of biological functions in the brain during normal aging might contribute to a better understanding of the pathogenesis of these diseases. We found that proteins displaying age-related alterations in rhythmicity in the hippocampus of mice were involved in various hallmarks of aging, including mitochondrial dysfunction, genomic instability, epigenetic alterations, and loss of protein homeostasis. A recent study identified age-dependent changes in dynamics of the oxidative stress response in the hippocampus of aged mice (Lacoste et al., 2017), consistent with our results. We also found rhythmic hippocampal proteins involved in the pathogenesis of several aging-associated neurodegenerative diseases affecting the brain, indicating that age-related alterations in circadian regulation are associated with and might contribute to the pathogenesis of these diseases.

Age-dependent changes in the temporal regulation of proteins involved in local hippocampal clock function, such as proteins involved in regulating protein phosphorylation and turnover, provide novel potential mechanisms through which aging may be associated with alterations in physiological circadian rhythms. Additional studies are needed to characterize the effects of aging on the circadian rhythms of post-translational modifications of proteins, given that our results implicate enzymes regulating protein acetylation and phosphorylation in hallmarks of aging and age-related functional decline. This is plausible given that we and others have previously shown the relevance of rhythmic protein phosphorylation in the hippocampus of young mice (Chiang et al., 2017; Snider et al., 2018).

Our study represents the first large-scale proteomic analysis of aging in mammals from a circadian perspective, and our findings provide a framework for understanding the links between age-related cognitive decline, neurodegenerative disorders, and the circadian clock. Circadian disruption is associated with hippocampal-dependent memory impairment, and it is conceivable that aging-induced alterations in the circadian regulation of processes critical for hippocampal function could contribute to this decline. Our results build upon previous studies examining the circadian proteomes of brain, liver, and heart tissues from young mice (Millius and Ueda, 2017) and the circadian transcriptomes of various central and peripheral tissues from young mice (Zhang et al., 2014) and baboons (Mure et al., 2018). Furthermore, a growing number of recent studies are exploring the effects of aging (Sato et al., 2017), environmental and genetic circadian disruption (Archer et al., 2014; Martino and Young, 2015), and various diets (Sato et al., 2017; Tognini et al., 2017) on the circadian regulation of cellular functions in humans and mice. Our dataset may therefore also serve as a resource to the circadian biology community for future studies investigating the effects of environmental and genetic modifications or potential therapeutic interventions on hippocampal function in mammals.

## Data Availability Statement

The mass spectrometry proteomics data have been deposited to the ProteomeXchange Consortium via the PRIDE partner repository with the dataset identifier PXD013364.

## Ethics Statement

The animal study was reviewed and approved by the University of Ottawa Animal Care Committee.

## Author Contributions

H-YC and DF conceived the study. C-KC, JM, ZN, XZ, BX, H-YC, and DF designed the study. PA, C-KC, JM, ZN, XZ, BX, and H-YC performed the experiments. PA, C-KC, ZN, and XZ analyzed the data. PA, JM, ZN, C-KC, XZ, BX, and DF interpreted the data. PA drafted the manuscript. All authors edited and approved the manuscript.

## Conflict of Interest

The authors declare that the research was conducted in the absence of any commercial or financial relationships that could be construed as a potential conflict of interest.
