# Supplementary material for: Aging Disrupts the Circadian Patterns of Protein Expression in the Murine Hippocampus
Source: Front Aging Neurosci. 2020 Jan 15;11:368. doi: 10.3389/fnagi.2019.00368 (PMC6974521; doi:10.3389/fnagi.2019.00368)
Supplement: Supplementary file 1 [file Data_Sheet_1.PDF]

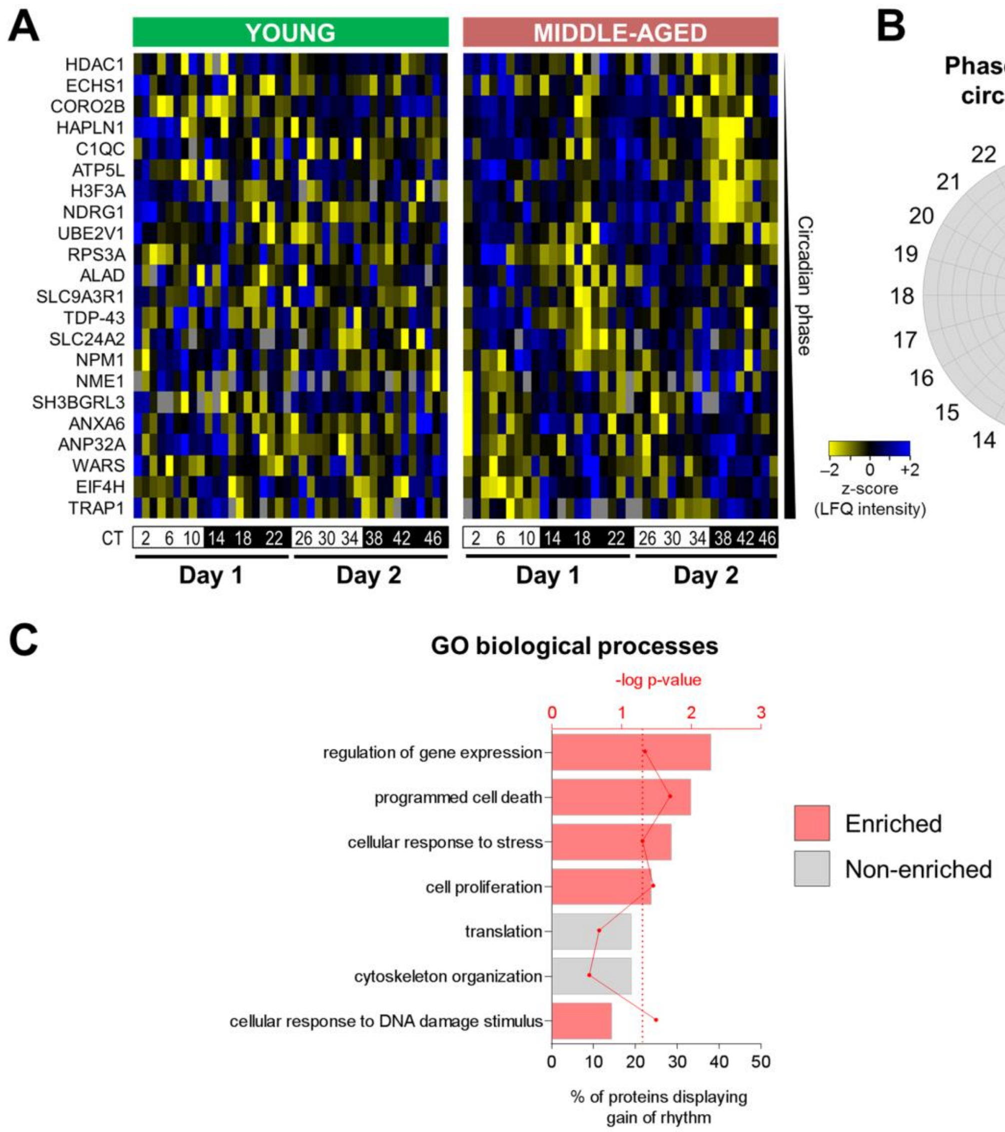

Supplementary Figure 1. Proteins gaining circadian rhythmicity in the hippocampus of middle-aged mice. (A) Heatmaps displaying z-score normalized abundances ($\log_{10}$ LFQ intensities) of circadian proteins specific to middle-aged mice, and their temporal expression profiles in young mice (*Left*) and middle-aged mice (*Right*). Circadian proteins were detected using the Perseus periodicity algorithm (period = 23.6 h; q-value < 0.25). **(B)** Phase distribution (enlarged from Figure 1F). **(C)** Functional annotation and enrichment analysis. The vertical red line denotes the cutoff for significantly enriched biological processes ($p \leq 0.05$, Fisher's exact test relative to background of accurately quantified proteins in our dataset).
