# Supplementary material for: Aging Disrupts the Circadian Patterns of Protein Expression in the Murine Hippocampus
Source: Front Aging Neurosci. 2020 Jan 15;11:368. doi: 10.3389/fnagi.2019.00368 (PMC6974521; doi:10.3389/fnagi.2019.00368)
Supplement: Supplementary file 3 [file Data_Sheet_3.PDF]

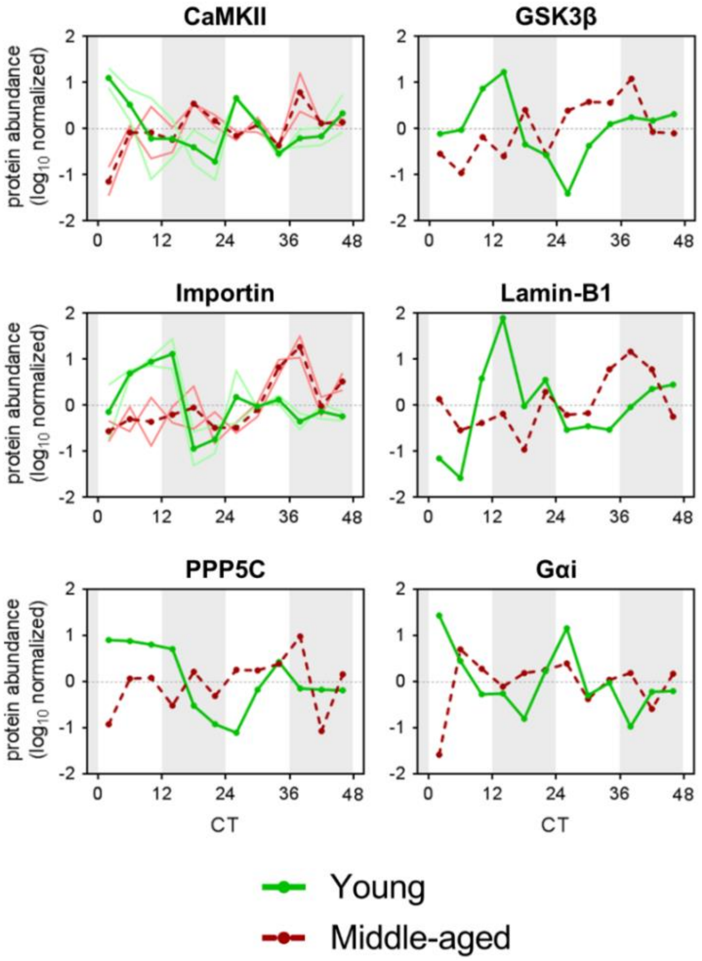

Supplementary Figure 3. Aging disrupts the circadian oscillations of proteins involved in clock regulation. Individual examples of rhythmic proteins involved in clock regulation and losing their circadian rhythms in abundance in the hippocampus of middle-aged mice.
