# Supplementary material for: Aging Disrupts the Circadian Patterns of Protein Expression in the Murine Hippocampus
Source: Front Aging Neurosci. 2020 Jan 15;11:368. doi: 10.3389/fnagi.2019.00368 (PMC6974521; doi:10.3389/fnagi.2019.00368)
Supplement: Supplementary file 2 [file Data_Sheet_2.PDF]

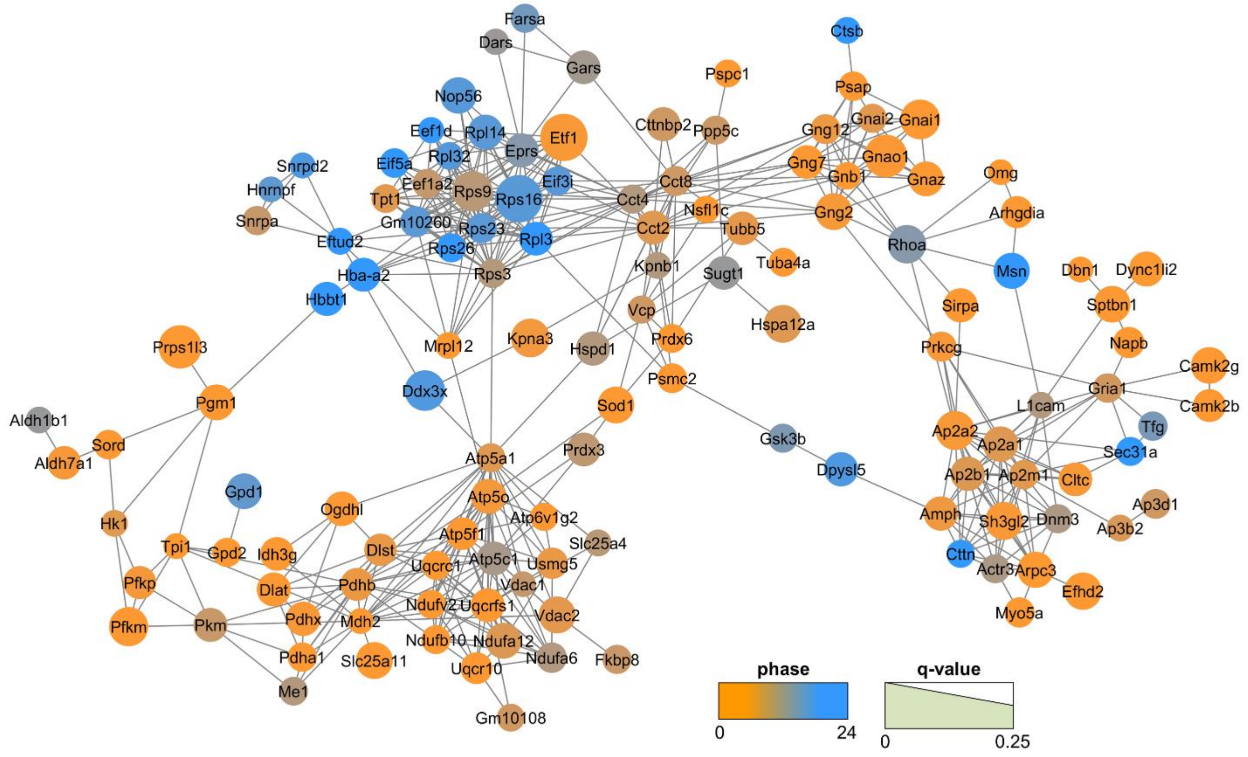

Supplementary Figure 2. Protein interaction network analysis of proteins displaying loss of circadian rhythmicity in the hippocampus during aging. Protein–protein interaction network of proteins displaying loss of circadian rhythmicity with aging. Protein interactions were determined using the STRING database (confidence cutoff = 0.7) and visualized using Cytoscape.
